# Outcomes of Low-Intensity Treatment of Acute Lymphoblastic Leukemia at Butaro Cancer Center of Excellence in Rwanda

**DOI:** 10.1200/JGO.2017.009290

**Published:** 2017-10-30

**Authors:** Fidel Rubagumya, Mary Jue Xu, Leana May, Caitlin Driscoll, Frank Regis Uwizeye, Cyprien Shyirambere, Katherine Larrabee, Alexandra E. Fehr, Umuhizi Denis Gilbert, Clemence Muhayimana, Vedaste Hategekimana, Shekinah Elmore, Tharcisse Mpunga, Molly Moore, Lawrence N. Shulman, Leslie Lehmann

**Affiliations:** **Fidel Rubagumya**, Muhimbili University of Health and Allied Sciences, Dar es Salaam, Tanzania; **Mary Jue Xu**, University of California, San Francisco, San Francisco, CA; **Leana May**, Children’s Hospital Colorado, Aurora, CO; **Caitlin Driscoll**, Mount Sinai Medical School, New York, NY; **Frank Regis Uwizeye**, **Cyprien Shyirambere**, and **Alexandra E. Fehr**, Inshuti Mu Buzima/Partners In Health; **Umuhizi Denis Gilbert**, **Clemence Muhayimana**, **Vedaste Hategekimana**, and **Tharcisse Mpunga**, Ministry of Health, Kigali, Rwanda; **Katherine Larrabee**, Geisinger Commonwealth School of Medicine, Scranton; **Lawrence N. Shulman**, University of Pennsylvania, Philadelphia, PA; **Shekinah Elmore**, Harvard Radiation Oncology Program; **Leslie Lehmann**, Dana Farber Cancer Institute; Boston Children’s Hospital, Boston, MA; and **Molly Moore**, University of Vermont, Burlington, VT.

## Abstract

**Purpose:**

Children with acute lymphoblastic leukemia (ALL) in low-income countries have disproportionately lower cure rates than those in high-income countries. At Butaro Cancer Center of Excellence (BCCOE), physicians treated patients with ALL with the first arm of the Hunger Protocol, a graduated-intensity method tailored for resource-limited settings. This article provides the first published outcomes, to our knowledge, of patients with ALL treated with this protocol.

**Methods:**

This is a retrospective descriptive study of patients with ALL enrolled at BCCOE from July 1, 2012 to June 30, 2014; data were collected through December 31, 2015. Descriptive statistics were used to calculate patient demographics, disease characteristics, and outcomes; event-free survival was assessed at 2 years using the Kaplan-Meier method.

**Results:**

Forty-two consecutive patients with ALL were included. At the end of the study period, 19% (eight) were alive without evidence of relapse: three completed treatment and five were continuing treatment. Among the remaining patients, 71% (30) had died and 10% (four) were lost to follow-up. A total of 83% (25) of the deaths were disease related, 3% (one) treatment-related, and 13% (four) unclear. Event-free survival was 22% (95% CI, 11% to 36%), considering lost to follow-up as an event, and 26% (95% CI, 13% to 41%) if lost to follow-up is censored.

**Conclusion:**

As expected, relapse was the major cause of failure with this low-intensity regimen. However, toxicity was acceptably low, and BCCOE has decided to advance to intensity level 2. These results reflect the necessity of a data-driven approach and a continual improvement process to care for complex patients in resource-constrained settings.

## BACKGROUND

Acute lymphoblastic leukemia (ALL) is the most common pediatric cancer worldwide.^1^ In high-income countries, survival rates have drastically improved from < 30% to 90% in the past 50 years.^2-5^ However, therapy remains challenging: most children require 2 to 3 years of ongoing therapy, and intensive supportive care is often needed during the early phases. In low- and middle-income countries (LMICs), conversely, survival rates remain poor—often < 35%.^6-10^ This reflects many challenges, including gaps in implementation of care, delayed or incorrect diagnosis, comorbid conditions, lack of needed treatment components, increased relapse rates, abandonment of therapy, death from toxicity, and suboptimal supportive care.^1,8,11,12^

Monitoring ALL outcomes can provide a useful metric of a program’s capacity to address delivery of complex longitudinal oncology care. Successful models for treating ALL in resource-limited settings have been reported in South America. For example, 5-year event-free survival (EFS) rates in a Brazilian hospital increased from 32% to 63% from 1980 to 2002 because of improvements in clinician training, patient social support, supportive care, and treatment availability and standardization.^13^ Data on similar models in sub-Saharan Africa are limited, but a Tanzanian cohort of 81 patients following the United Kingdom Acute Lymphoblastic Leukaemia 2003 (UKALL2003) protocol had a 2-year EFS rate of 26%.^14^ Obstacles included availability and affordability of chemotherapy and supply of blood products.

Located in northern rural Rwanda, Butaro Cancer Center of Excellence (BCCOE) is housed in the Ministry of Health’s Butaro District Hospital and provides free cancer care in partnership with Partners In Health/Inshuti mu Buzima and the Dana-Farber/Brigham and Women’s Cancer Center. BCCOE has been described in greater detail elsewhere.^15,16^ After a national consensus meeting in March 2012, BCCOE began treating patients with ALL in accordance with the graduated intensity regimen proposed by Hunger et al,^17^ an approach developed specifically for low-resource settings. Treatment facilities begin with regimen 1, a low-intensity medication regimen, and advance to an increased medication regimen only after demonstrating that treatment-related toxicity is acceptably low (less than one death for every 25 patients). To our knowledge, no prior studies have reported on this regimen in a low-resource setting. Therefore, the objective of this study is to report the outcomes of using regimen 1 of the Hunger protocol on pediatric patients at BCCOE, as well as the quantitative measures of resource demands and delays in care.

## METHODS

### Setting and Treatment

During the study period, BCCOE treated 169 pediatric oncology patients, in whom ALL was the second most common diagnosis (after nephroblastoma). At the time of this study, BCCOE offered patients with ALL basic imaging (x-ray and ultrasound), laboratory tests, bone marrow biopsy, and pathology processing. In addition, social services covered costs for transportation and nutritional support. Pathologists at Brigham and Women’s Hospital (Boston, MA) or Rwandan referral hospitals or visiting pathologists at BCCOE interpreted tissue specimens.

BCCOE used regimen 1 of the Hunger protocol, composed of vincristine, prednisone, cyclophosphamide, intrathecal methotrexate, 6-mercaptopurine, dexamethasone and l-asparaginase (Appendix Table A1). There is no anthracycline administered in level 1 of this protocol. Given health system limitations, patients were not uniformly evaluated for CNS involvement, bone marrow response to therapy, or prednisone response, factors often required for clinical risk stratification, but in regimen 1 all patients receive identical therapy. Most patients remained continuously hospitalized during induction and consolidation, given the frequent chemotherapy doses and associated adverse effects. On-site visiting Dana-Farber/Brigham and Women’s Cancer Center nurses trained Rwandan nurses in chemotherapy preparation and management of patients with cancer. Radiotherapy was not included in the protocol, and currently there is no radiotherapy care available in Rwanda.

### Data Management and Analysis

Data were collected for consecutive patients with ALL presenting at BCCOE from July 1, 2012 to December 31, 2015. Patients were identified and data were collected using the electronic medical records system OpenMRS; additional data were collected from patient charts using a structured chart abstraction form. Analysis was performed using STATA v12 (StataCorp, College Station, TX). This study was approved by the Rwanda National Ethics Committee, the Inshuti Mu Buzima Research Committee, and the Institutional Review Board at Partners Healthcare, Boston, Massachusetts.

Patients were considered to start a phase of treatment once the first chemotherapy agent was administered. A phase of treatment was considered complete if documented in the medical record. Documented treatment delays were those that postponed chemotherapy administration for any duration. Disease-related deaths were defined as occurring either before treatment began or after relapsed or refractory disease. Relapse was confirmed by clinical symptoms, derangement of CBC, and presence of blasts in the peripheral blood film after a period of remission. Refractory disease was defined as failure to achieve remission after completion of either induction or consolidation. The remaining deaths were deemed either treatment related (those after initiation of chemotherapy) or unclear (treatment failure clinically suspected but not confirmed before death). Loss to follow-up (LTFU) was strictly defined as missing the most recent appointment.

EFS from intake for all patients diagnosed with pathology was assessed at 2 years using the Kaplan-Meier method. This was calculated twice. First, events were death from any cause, relapsed disease, and LTFU. Second, events were death from any cause and relapsed disease; LTFU was right-censored.

## RESULTS

### Patient Demographics and Disease Characteristics

Fifty-four patients were evaluated or treated for ALL from July 1, 2012 to June 30, 2014. Diagnoses of eight patients were not pathologically confirmed before exiting the program, three patients had prior treatment presenting with relapsed or residual disease, and one patient was treated with an alternative protocol because of stroke. The remaining 42 patients had newly diagnosed, pathologically confirmed disease and were started on level 1 of the Hunger protocol at BCCOE.^17^ Forty-two patients were evaluated with a new pathologically confirmed diagnosis of ALL (Tables 1 and 2). Median age was 10 years (range, 0.38 to 40.35 years), and 55% (23) of patients were male. Eighty-six percent (36) of patients lived in Rwanda, and 14% (six) lived in Burundi (Table 3). Seventy-nine percent were HIV negative, 5% were positive, and 17% had unknown HIV status. Seventy-four percent (31) were transferred to Butaro from national referral facilities. Before arriving at BCCOE, 29% (12) of patients received allopurinol and steroids (17% [seven] steroids, 7% [three] allopurinol), and 48% (20) of patients received no prior cancer-directed treatment.

**Table 1:**
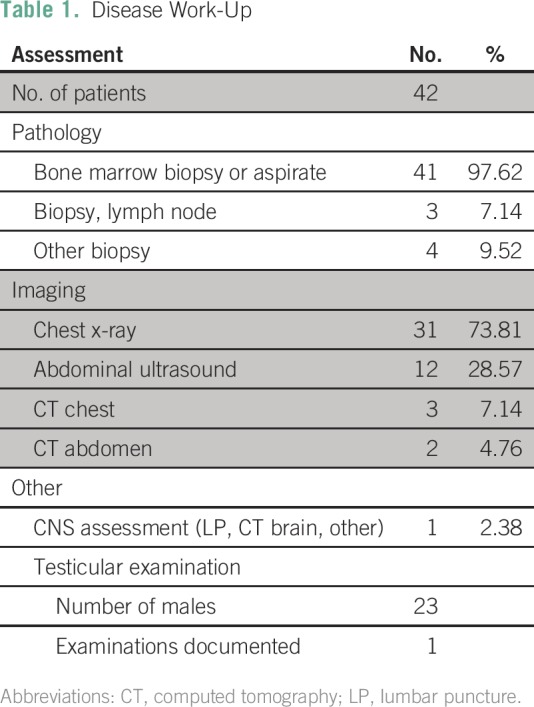
Disease Work-Up

**Table 2:**
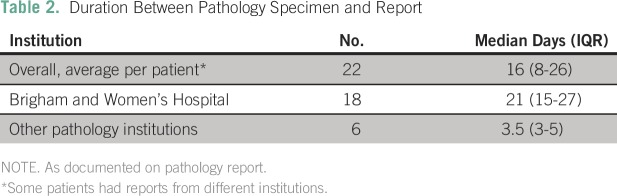
Duration Between Pathology Specimen and Report

**Table 3:**
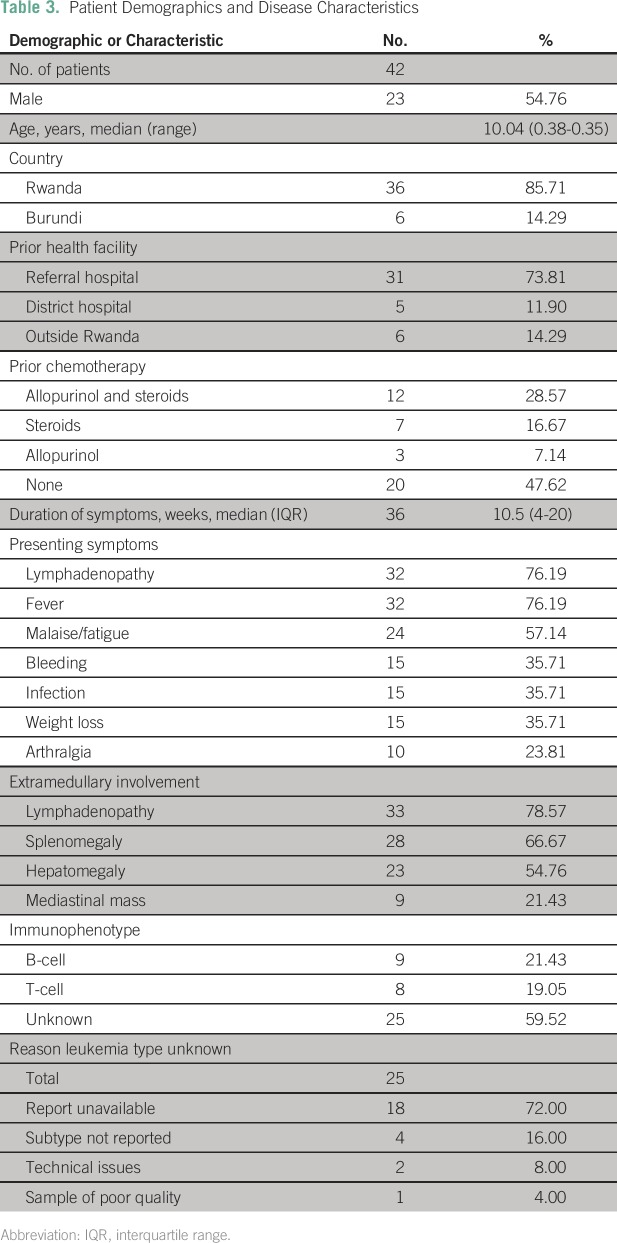
Patient Demographics and Disease Characteristics

Patients presented to BCCOE a median of 10.5 weeks (interquartile range [IQR], 4-20 weeks) after onset of symptoms, the most common of which were lymphadenopathy 76% (32), fever 76% (32), and malaise 57% (24). The most common extramedullary sites of involvement were lymph nodes 80% (33), spleen 67% (28), and liver 55% (23).

Disease immunophenotype was unknown for 60% (25) of patients, 21% (nine) had B-cell, and 19% (eight) had T-cell. In addition to subtype, other information, such as CNS involvement, was often unavailable (Tables 1 and 2). Using the limited information available for stratification, > 75% of patients in the study would have been classified as high or very high risk (Appendix Table A5).

### Treatment and Outcomes

**Fig 1:**
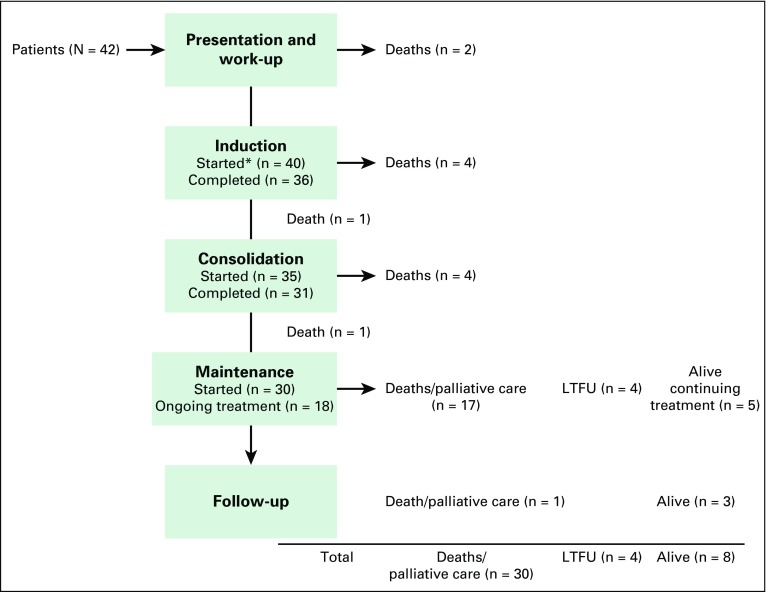
Treatment and patient events. (*) Started phase of induction, consolidation, and maintenance defined as having received chemotherapy. LTFU, lost to follow-up.

Of the 42 patients who began therapy for ALL, 95% (40) initiated induction, 83% (35) consolidation, and 71% (30) maintenance (Fig 1). At the end of the analysis period, 19% (eight) of patients were alive without evidence of relapse: three completed treatment and were in follow-up and five were still receiving treatment. Seventy-one percent (30) had died, and 10% (four) were LTFU (Table 4). When LTFU was considered an event, estimated 2-year EFS was 22% (95% CI, 11% to 36%); when LTFU was right-censored, estimated 2-year EFS was 26% (95% CI, 13% to 41%). Overall, the median time from enrollment at BCCOE to time of event was 9 months (IQR, 2-19 months).

**Table 4:**
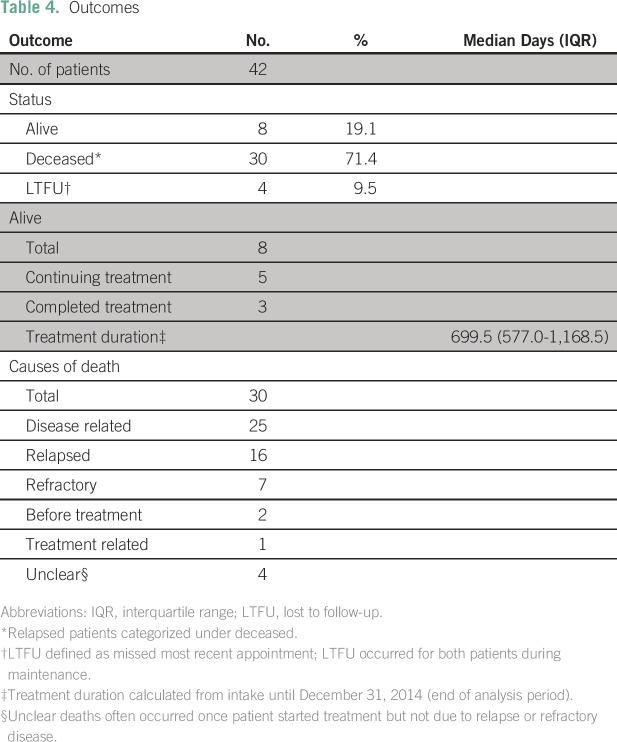
Outcomes

**Fig 2:**
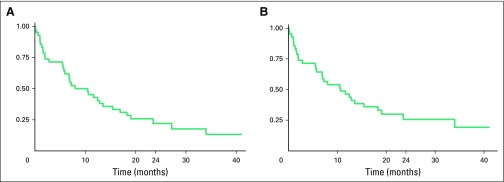
Censored event-free survival (EFS; N = 42). (A) Estimated 2-year EFS lost to follow-up (LTFU) as event: 22% (95% CI, 11% to 36%). (B) Estimated 2-year EFS LTFU censored: 26% (95% CI, 13% to 41%).

For patients alive at time of analysis, treatment duration was a median of 699.5 days (IQR, 577-1,168.5 days). Of the 30 deaths, 83% (25) were disease related (16 relapsed, seven were refractory, and two died before treatment initiation), 3% (one) were treatment-related, and 13% (four) were unknown. Deaths occurred throughout all phases of treatment, although concentrated in two periods: within the first 2 months after presentation, and 6 to 8 months after initiation of therapy, most frequently during the first cycles of maintenance (Fig 2).

### Resource Demands of Treatment

**Fig 3:**
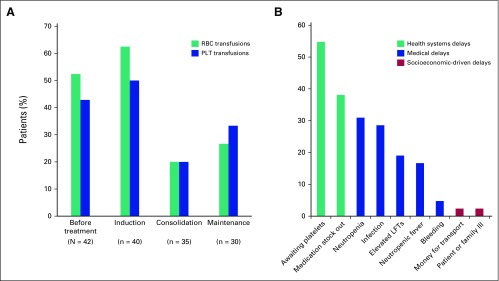
Treatment-related resource demands. (A) Patients requiring blood products. (B) Causes of chemotherapy delay (N = 42). LFTs, liver function tests; PLT, platelet.

Even with this low-intensity approach, many resources were required to support these patients with ALL (Appendix Table A2). For the 42 patients evaluated before initiating therapy, 52% (22) required packed red blood cells and 43% (18) required platelets. Throughout, the median hemoglobin was 8.3 g/dL (IQR, 7.7-9.8 g/dL; n = 31) and the median platelet level was 25.5 × 10^3^/μL (IQR, 12-51 μL; n = 30). For the 40 patients who started induction therapy, 63% (25) required packed red blood cells and 50% (20) required platelets (Fig 3).

The most common cause of treatment delay was thrombocytopenia, present in 55% (23) of patients (Fig 3), and delayed platelet availability as products were transported from blood banks at offsite locations. Fluctuations in supply of two chemotherapy drugs, l-asparaginase and methotrexate, led to rescheduling of treatment cycles affecting care in 38% (16) of patients (Appendix Table A3). Medical-related delays included infections, neutropenia, elevated liver transaminases, neutropenic fever, and bleeding. Of note, delays resulting from socioeconomic barriers to care were few (one from lack of money for transport and one from illness of the patient or family member). Socioeconomic-related delays, however, were likely not fully captured in this retrospective review.

## DISCUSSION

ALL is the most common hematologic malignancy in children,^1^ and the ability to provide care for patients with ALL is an essential component of oncology programs serving LMICs. However, given the duration of therapy, the recurrent periods of neutropenia, and the supportive care requirements, including transfusions and antibiotics, delivery of care requires a robust medical infrastructure. To our knowledge, our results represent the first published outcomesfrom a rural cancer center in a low-income country using the strategy proposed by the Hunger group^17^ that restructures treatment into stratified levels of therapeutic intensities. This model recommends an initial, low-intensity regimen and data capture to assess the incidence of treatment-related deaths. Once care can be demonstrated to be safely provided, intensity of care can be increased.

We piloted this approach at BCCOE, a rural-based cancer center where care is provided by pediatricians, internists and general practitioners follow strict treatment protocols, and there is support from visiting on-site oncologists and regular remote support from affiliated oncologists. As expected, given the initial low-intensity and anthracycline-free regimen, relapse was the major cause of treatment failure and led to survival rates similar to the 26% estimated 2-year EFS and 8.1 month median survival of a Tanzanian cohort.^14^ In North American cohorts, an estimated 5% to 25% of patients with ALL receive cranial radiation for treatment and prophylaxis of CNS lymphoma.^18^ In our patient cohort, 53% of patients experienced relapse. Given this high relapse rate in patients receiving low-intensity treatment, it is likely the low-intensity treatment was insufficient for long-term survival. For some critically ill patients, the precise cause of mortality was difficult to determine when signs of infection coincided with treatment initiation. Nevertheless, definitive treatment-related toxicity was sufficiently low to advance to the next level of therapy per Hunger guidelines.^17^

Intensification of treatment, however, requires disease stratification, a challenge given the limited number of physicians, inconsistent access to CSF diagnostics, difficulties in reliably obtaining immunophenotyping, and delays in pathology reports (Tables 1 and 2).^8,14^ In addition, the lack of in-country radiation therapy poses financial and operational challenges. When disease stratification can be achieved along with simultaneous training of hospital personnel, strengthening of supportive care, and standardizing of treatment regimens, outcomes can markedly improve, as was seen with the 63% 5-year EFS in Brazil.^13^ This data-driven approach to improving care can only be achieved in the context of collecting and analyzing high-quality patient data, a challenge in all health care settings and particularly in a resource-constrained environment.

Treatment abandonment, often cited as a cause of treatment failure for patients with ALL, was uncommon at BCCOE. Although additional follow-up will be needed, the 10% lost to follow-up rate was modest compared with 35% in Indonesia^19^ and 22% in El Salvador.^8^ Patient social support, such as coverage of transportation and chemotherapy costs as provided at BCCOE, have helped in similar settings and have led to lower abandonment rates of 9% in Tanzania^14^ and 0.5% in Brazil.^13^ Given its mission to provide care to all patients, both social and clinical, BCCOE has also noted low levels of abandonment and delays in treating other cancers, such as nephroblastoma.^20^

In the presented approach to classifying delays, health system delays, such as waiting for blood products and availability of chemotherapy agents, were the most common in our patient population. Inconsistent sources for both blood products and some chemotherapy (Appendix Table A3) were major challenges. An estimated 8 million units of blood are needed in sub-Saharan African countries annually, and only 3 million units are collected.^21^ At BCCOE, > 40% of patients who started treatment required transfusions; this drastically underscores the importance of a reliable system to provide supportive clinical care.^13,14,22^ Quantitatively documenting this need could serve as a tool to predicting and planning for future transfusion needs in similar settings. Some minor lapses in availability of chemotherapeutics led to additional delays. Alterations in chemotherapy regimens because of lack of drug availability have led to poorer survival in both resource-rich and resource-constrained settings,^14,23^ and, therefore, more accurate predictions and a reliable supply chain for ALL medications and transfusions has become a crucial goal at BCCOE.

In the context of Rwanda’s dedication to providing cancer care, the Rwandan Ministry of Health has hosted regular national consensus meetings for cancer protocol development. The BCCOE clinical team presented these data at the pediatric protocol meeting in the spring of 2015. After reviewing the results, the committee supported intensifying the national ALL treatment protocol, given the high relapse rate and acceptable treatment-related death rate. This data-driven approach that focuses particularly on resource demands of care is critical to patient outcomes in this and other resource-constrained settings.

In conclusion, this study details our experience treating patients with ALL in a rural Rwandan cancer center and to our knowledge reports the first published outcomesusing the lowest intensity level of the Hunger ALL protocol. As expected with a low-intensity regimen, a high rate of disease-related mortality occurred, interestingly clustering in two time periods. However, treatment-related toxicity was below the threshold suggested for increasing treatment intensification. In addition to supplementing the limited literature on ALL care in sub-Saharan Africa, the quantification of transfusion needs and classification of treatment delays can be used to predict challenges to care in similar settings.

Overall, we have demonstrated that an iterative model of cancer care, delivered by nononcologists with remote oncological support, where implementation is followed by analysis of outcomes and subsequent evidence-based changes for improvement of care, allows for accountable delivery of ALL treatment in LMICs using the Hunger approach. We are now risk-stratifying patients and advancing to regimen 2 for high-risk patients after an intensive educational program for providers. These results point to the necessity of a data-driven approach to optimize care for complex patients in resource-constrained settings.

## Figures and Tables

**Table A1:**
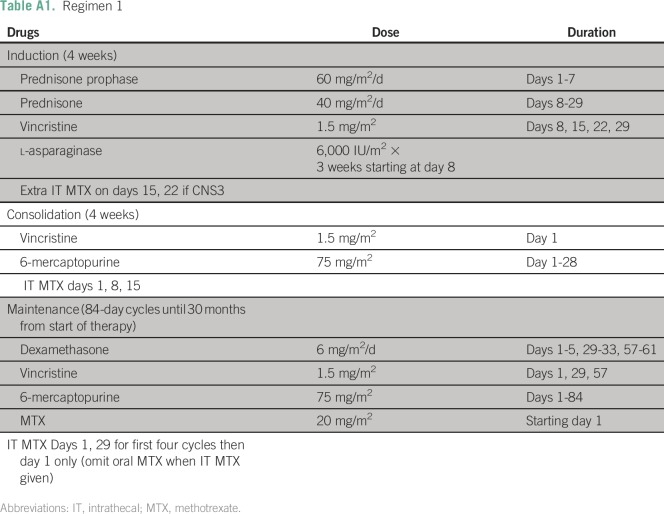
Regimen 1

**Table A2:**
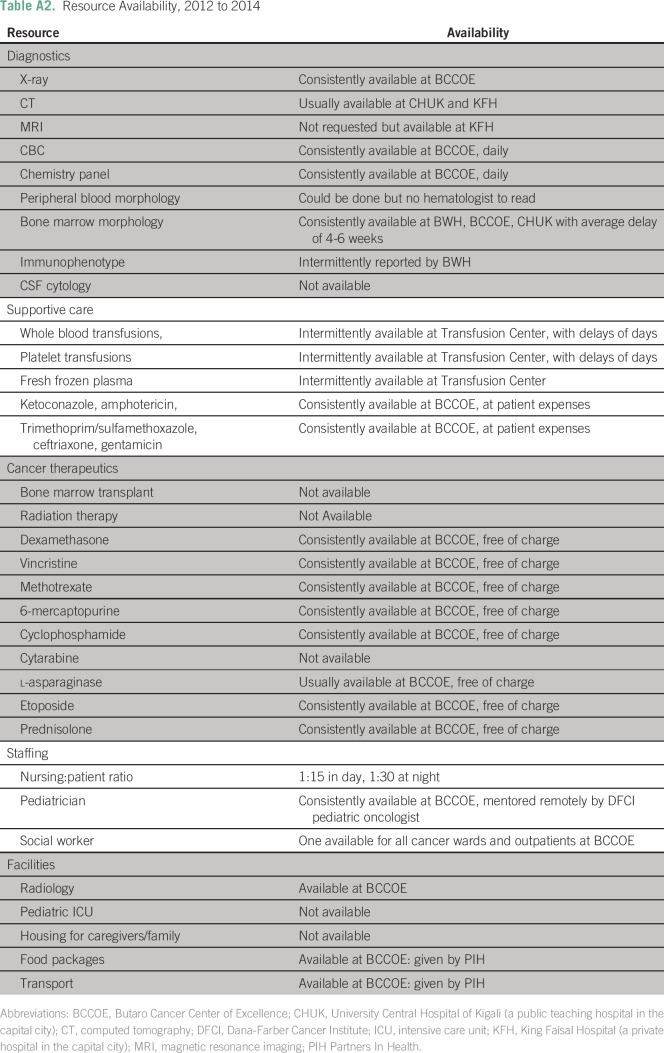
Resource Availability, 2012 to 2014

**Table A3:**
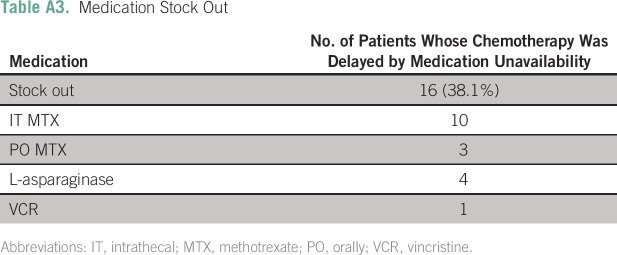
Medication Stock Out

**Table A4:**
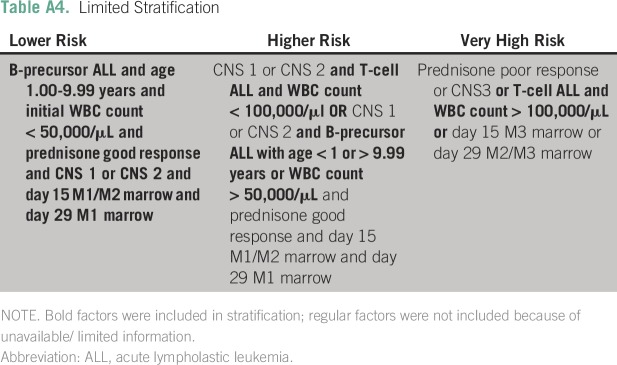
Limited Stratification

**Table A5:**
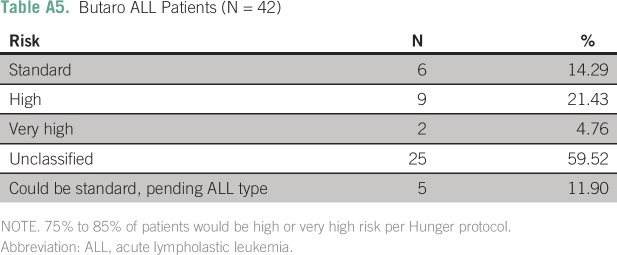
Butaro ALL Patients (N = 42)
